# Patient-Derived iPSC Modeling Reveals Dysregulation of the Mucin-Type O-Glycan Biosynthesis-Related Transcriptional Program in *TBX6*-Associated Congenital Scoliosis

**DOI:** 10.3390/cells15191785

**Published:** 2026-09-30

**Authors:** Jing Hao, Zhengye Zhao, Shuang Li, Di Liu, Guozhuang Li, Lin Wang, Jingyi Xie, Zhifa Zheng, Jianguo Zhang, Zhihong Wu, Nan Wu, Lina Zhao

**Affiliations:** 1 Department of Orthopedic Surgery, Peking Union Medical College Hospital, Chinese Academy of Medical Sciences & Peking Union Medical College, Beijing 100730, China; 2 Department of Orthopedic Surgery, Peking University First Hospital, Beijing 100034, China; 3 Stem Cell Facility, National Infrastructures for Translational Medicine, Peking Union Medical College Hospital, Chinese Academy of Medical Sciences & Peking Union Medical College, Beijing 100730, China; 4 State Key Laboratory of Complex Severe and Rare Diseases, Peking Union Medical College Hospital, Chinese Academy of Medical Sciences & Peking Union Medical College, Beijing 100730, China

**Keywords:** *TBX6*, congenital scoliosis, induced pluripotent stem cells, mucin-type O-glycan biosynthesis, osteochondral differentiation

## Abstract

TBX6-associated congenital scoliosis (TACS) is a genetic subtype of congenital scoliosis caused by reduced *TBX6* gene dosage, most commonly through compound inheritance of a rare loss-of-function allele and a hypomorphic T-C-A haplotype. However, the mechanisms linking TBX6 dysfunction to impaired human skeletal development remain unclear. Here, we established a patient-derived induced pluripotent stem cell (iPSC) model of TACS and recapitulated key stages of axial skeletal development through stepwise differentiation into paraxial mesoderm, nascent somites, sclerotome, and subsequently osteogenic and chondrogenic lineages. TACS-derived cells showed impaired differentiation, with reduced mineralized matrix deposition and diminished cartilage matrix formation. Bulk transcriptomic profiling revealed marked alterations in both lineages. Notably, mu-cin-type O-glycan biosynthesis was consistently enriched among downregulated genes in osteogenic and chondrogenic cells and was also significantly represented among genes commonly downregulated across both lineages. qRT-PCR further confirmed reduced expression of *GALNT3*, *GALNT5*, *GALNT7*, *GALNT10*, *GCNT1*, and *ST6GALNAC2*, which participate in distinct steps of mucin-type O-glycan biosynthesis. Consistent with these transcriptional alterations, Tn-antigen staining showed reduced Tn-associated O-GalNAc signals in TACS-derived osteogenic and chondrogenic cells. In silico promoter analysis identified candidate sequences matching the TBX6-binding motif within the upstream regions of these genes, providing potential regulatory regions for future experimental investigation. Collectively, these results establish a human iPSC model for TACS and identify coordinated dysregulation of a mucin-type O-glycan biosynthesis-related transcriptional program, accompanied by reduced Tn-associated O-GalNAc signals, as molecular features associated with impaired osteochondral differentiation in this model. These findings should be interpreted within the context of a single family-based iPSC model, and validation in additional TACS lines and isogenic TBX6-corrected models will be required.

## 1. Introduction

Congenital scoliosis (CS) is a developmental spinal disorder characterized by structural vertebral malformations arising during embryogenesis. Normal formation of the vertebral column depends on the coordinated specification of paraxial mesoderm, periodic segmentation of the presomitic mesoderm into somites, and subsequent differentiation of somite-derived progenitors, including the sclerotome, into components of the axial skeleton [1]. Disruption of these developmental processes can therefore lead to congenital vertebral malformations. Among the transcriptional regulators governing paraxial mesoderm development and somitogenesis, T-box transcription factor 6 (*TBX6*) has emerged as a key determinant of axial skeletal development. Loss of Tbx6 profoundly disrupts paraxial mesoderm specification, causing cells normally destined for a mesodermal fate to instead give rise to ectopic neural structures [2]. In addition to directing paraxial mesoderm identity, *TBX6* participates in the neural mesodermal fate decision by modulating *SOX2* expression, thereby reinforcing its critical function as an essential regulator of early axial mesoderm development [3]. *TBX6* function undergoes strict spatiotemporal regulation during mesoderm specification and somite morphogenesis. In pluripotent stem cell and embryonic models, Sadahiro et al. demonstrated that Tbx6 elicits nascent mesoderm formation, and the duration of its expression dictates the diversification into distinct mesodermal lineages, whereby sustained Tbx6 activity favors somite-associated differentiation [4]. Beyond this, Tbx6 governs the molecular transition from presomitic mesoderm to morpho-logically defined somites: Ripply2-dependent Tbx6 degradation proves essential for accurate segment-border formation during murine somitogenesis [5], while recent evidence reveals that Ripply-mediated Tbx6 repression converts oscillatory segmentation-clock activity into stable somite boundaries [6]. Recent synthesis of vertebrate somitogenesis further emphasizes the importance of precisely coordinated temporal, spatial, and signaling dynamics during presomitic mesoderm segmentation and somite morphogenesis [7]. Collectively, these findings indicate that normal axial development relies on finely tuned *TBX6* activity, rather than its mere presence or absence.

The developmental importance of *TBX6* is strongly supported by human genetic studies. Wu et al. uncovered a compound inheritance model wherein a rare *TBX6* loss-of-function allele acts in trans with a prevalent hypomorphic T-C-A haplotype. This combinatorial genetic pattern diminishes functional *TBX6* dosage and explains approximately 11% of congenital scoliosis cases in the original patient cohort [8]. This genetic paradigm has since been replicated in an independent Japanese population [9]. Genotype–phenotype analyses further established *TBX6*-associated congenital scoliosis (TACS) as a clinically distinguishable subtype of CS, characterized by recurrent vertebral phenotypes and supporting a dosage-dependent disease model [10]. Importantly, the relationship between *TBX6* dosage and vertebral development is bidirectional: increased *TBX6* dosage can also induce congenital cervical vertebral malformations in humans and mice [11]. More recently, large-scale exome/genome analysis of 873 individuals with congenital vertebral malformations identified *TBX6* among genes carrying significant ultra-rare variant burdens and, through integration with single-nucleus transcriptomic profiles of the developing human spine, further reinforced the developmental context of genetic susceptibility to vertebral malformations [12]. Clinical studies have also begun to demonstrate the translational relevance of molecularly defining TACS, including its potential association with distinct long-term surgical outcomes [13]. Together, these genetic, developmental, and clinical observations establish TBX6 dosage as an important determinant of normal vertebral development and underscore TACS as a biologically and clinically meaningful subtype of congenital scoliosis.

Despite these advances, an important mechanistic gap remains between altered *TBX6* dosage and the structural skeletal abnormalities ultimately observed in patients with TACS. Most experimental studies of *TBX6* have focused on early developmental events, including mesoderm specification, neural mesodermal fate determination, segmentation-clock regulation, and somite boundary formation [2,3,4,5,6,7]. These studies have substantially advanced our understanding of the role of *TBX6* in early somitogenesis, but considerably less is known about how disease-associated *TBX6* dysfunction affects subsequent development of human somite- and sclerotome-derived skeletal lineages. This distinction is important because vertebral development continues well beyond somite segmentation: sclerotomal cells undergo migration and condensation and subsequently contribute to cartilage formation and endochondral ossification during vertebral morphogenesis [1]. In particular, whether reduced *TBX6* activity disrupts molecular programs required for osteogenic and chondrogenic differentiation remains poorly understood. Addressing this question is important because the clinical manifestations of TACS ultimately arise from structural abnormalities of vertebral cartilage and bone, whereas the molecular events linking early *TBX6* dysfunction to these later developmental defects have not been fully defined.

Human pluripotent stem cell-based models provide an opportunity to bridge this developmental gap. Because *TBX6* functions in a highly stage-dependent manner, analyses of mature skeletal cells alone cannot readily reconstruct the sequential developmental consequences of a germline *TBX6* defect. Recent advances have demonstrated that human pluripotent stem cells can recapitulate key stages of paraxial mesoderm and somite development in vitro. Three-dimensional paraxial mesoderm organoids can recapitulate molecular and morphological features of human somitogenesis and generate somite derivatives [14]. Complementary directed-differentiation approaches have shown that hPSCs can be sequentially induced through presomitic mesoderm, somite, and sclerotome-associated stages and subsequently generate osteogenic and chondrogenic derivatives [15]. Recent reviews of hPSC-based skeletal modeling further highlight the feasibility of reconstructing developmental trajectories from paraxial mesoderm toward axial skeletal lineages and applying these systems to human genetic skeletal disorders [16]. More recently, somitoid-based functional genomic studies have demonstrated the scalability of such models for interrogating human somitogenesis, with enhancer-associated profiling identifying developmental regulators including *MESP2* and *TBX6* [17]. Patient-derived induced pluripotent stem cells (iPSCs) offer the additional advantage of retaining the endogenous dis-ease-associated genotype within its native genetic background, enabling developmental abnormalities to be examined across sequential lineage transitions.

In the present study, we generated iPSC lines from peripheral blood mononuclear cells (PBMCs) obtained from a TACS patient carrying the pathogenic *TBX6* compound-inheritance genotype and his phenotypically unaffected mother. These cell lines underwent stepwise differentiation to recapitulate the developmental progression from paraxial mesoderm to nascent somites and sclerotome intermediates, followed by terminal osteogenic and chondrogenic differentiation. The somite differentiation protocol applied in this study was adapted from a previously established human somitogenesis induction system [15]. By integrating systematic developmental phenotyping with transcriptomic profiling of osteochondral lineages, we aimed to identify the core molecular programs driving defective skeletal differentiation, thereby constructing a mechanistic model that links *TBX6* insufficiency to pathological vertebral maldevelopment.

## 2. Materials and Methods

### 2.1. Study Participants and Ethical Approval

The use of iPSCs in this study was approved by the Ethics Committee of Peking Union Medical College Hospital (Approval No.2022SLCJ844) on 19 August 2022. The approved project title was “Research on Early Detection and Precise Intervention for Major High Impact and Teratogenic Skeletal Diseases”. All human samples were obtained with written informed consent from all donors, in accordance with the Declaration of Helsinki.

### 2.2. Genetic Analysis and Sanger Sequencing

Genomic DNA was extracted from peripheral blood mononuclear cells of the TACS proband and from the corresponding patient-derived TACS line and the asymptomatic maternal familial comparator (CTRL) line. The maternal line was used as an asymptomatic familial comparator rather than a genotype-neutral control, because the mother carries the hypomorphic T-C-A haplotype but not the patient’s TBX6 loss-of-function allele. The coding exons and flanking intronic regions of the *TBX6* gene were amplified by PCR, and purified amplicons were subsequently analyzed via Sanger sequencing to validate the *TBX6* compound-inheritance genotype in both primary patient samples and established iPSC lines. All sequencing procedures were professionally performed by Guodian (Beijing) Pharmaceutical Technology Co., Ltd. (Beijing, China), in accordance with standard operational protocols.

### 2.3. Generation and Culture of Patient-Derived iPSCs

Patient-derived iPSCs were generated by Guodian (Beijing) Pharmaceutical Technology Co., Ltd. Briefly, cryo-preserved PBMCs were thawed and seeded in 24-well plates with complete PBMC medium on day −4. Half-medium changes were performed daily from day −3 to day −1. On day 0, PBMCs were transduced overnight using the CytoTune™ 2.0 Sendai virus reprogramming kit. On day 1, cells were collected, centrifuged to remove residual virus, resuspended in complete PBMC medium and cultured for another 2 days. The medium was switched to cytokine-free PBMC medium on day 3, and transduced cells were replated onto vitronectin-coated plates. Cytokine-free PBMC medium was refreshed every other day from day 4 to day 6. Half-medium replacement with Essential 8™ medium was carried out on day 7. From day 8 to day 25, daily full-medium changes in Essential 8™ medium were applied, and iPSC colonies were monitored. Undifferentiated single iPSC colonies of suitable size were manually picked and transferred to Matrigel-coated plates. The iPSC lines were further expanded by serial passaging until stable, then cryopreserved.

### 2.4. IPSC Characterization

Pluripotency of patient-derived iPSCs was characterized by immunofluorescence staining, alkaline phosphatase staining, karyotype analysis and Sanger sequencing. For immunofluorescence staining of pluripotent markers, iPSCs cultured in 24-well plates were rinsed with DPBS and fixed in 4% paraformaldehyde. Fixed cells were permeabilized with permeabilization buffer at room temperature, followed by blocking with blocking solution. After two washes with DPBS, cells were incubated with primary antibodies at room temperature. Subsequently, cells were washed twice with DPBS and incubated with Anti-Mouse AF 488 and Anti-Rabbit AF 555 IgG-PE secondary antibodies. Cell nuclei were counterstained with Hoechst solution. After final DPBS rinses, cells were immersed in 1% BSA solution and subjected to fluorescence imaging. Alkaline phosphatase staining was performed to assess the undifferentiated status of iPSCs, which exhibited normal alkaline phosphatase activity. Chromosomal karyotype analysis confirmed the genomic stability and normal karyotype of iPSC lines. In addition, Sanger sequencing was performed to verify the genetic background of patient-derived iPSCs, and the experimental procedure was consistent with that described in Section 2.2.

### 2.5. Osteogenic and Chondrogenic Cell Induction and Staining Verification

Osteogenic and chondrogenic differentiation were performed following previously described protocols with minor modifications [15]. Two independently established iPSC clones from each donor were used for osteogenic and chondrogenic differentiation experiments. Major differentiation experiments were independently repeated three times. Unless otherwise specified, representative data are shown from experiments yielding consistent phenotypic findings across independent differentiation runs. Briefly, iPSCs were dissociated with TrypLE Express on day −1 and seeded onto Matrigel-coated plates at 25,000 cells/cm in mTeSR1 medium supplemented with 10 μM Y-27632. Somite differentiation was initiated on day 0 by culture in basal differentiation medium (BDM) containing 3 μM CHIR99021 for 2 days, followed by treatment with BDM supplemented with 200 nM LDN193189 and 10 μM SB431542 for another 2 days, with daily medium changes. For osteogenic differentiation, cells were subjected to the 4-day somite induction procedure, dissociated on day 4, and reseeded at 35,000 cells/cm in BDM containing 20 ng/mL FGF2 and 300 nM smoothened agonist for 2 days. On day 6, cells were cultured in StemPro Osteogenesis Differentiation medium for 28 days, with daily medium replacement before day 6 and every other day thereafter. For chondrogenic differentiation, cells received identical induction treatment for the first 6 days. On day 6, cells were digested, and 200,000 cells were resuspended in StemPro Chondrogenesis Differentiation medium, centrifuged at 300× *g* for 5 min to form pellets, and cultured for 28 days. Medium was refreshed daily before day 6 and every 3 days for the remaining culture period.

After osteogenic induction, extracellular mineralized matrix deposition was visualized by Alizarin Red S staining using commercial Alizarin Red S Solution (catalog No. ALIR-10001, Cyagen Biosciences, Suzhou, China). Briefly, differentiated osteogenic cells were rinsed with DPBS, fixed with 4% paraformaldehyde, and stained with Alizarin Red S working solution at room temperature according to the manufacturer’s instructions. Alcian Blue staining and immunofluorescence staining of paraffin-embedded chondrogenic pellet sections were performed by Wuhan Sevier Biotechnology (China) to evaluate cartilage-matrix deposition and the expression of chondrogenic markers.

### 2.6. RNA Extraction and qRT-PCR

Total RNA was extracted from cells using the FastPure^®^ Cell/Tissue Total RNA Isolation Kit V2 (RC102, Vazyme, Nanjing, China) according to the manufacturer’s instructions. RNA concentration and purity were assessed using a NanoDrop spectrophotometer (Thermo Fisher Scientific, Waltham, MA, USA). Reverse transcription was performed using the Hifair^®^ AdvanceFast 1st Strand cDNA Synthesis Kit (11150ES60, Yeasen Biotechnology, Shanghai, China) to synthesize complementary DNA (cDNA) from total RNA. Quantitative real-time PCR (qRT-PCR) was performed using Hieff^®^ qPCR SYBR^®^ Green Master Mix (11201ES08, Yeasen Biotechnology, Shanghai, China) on a LightCycler^®^ 480 II system (Roche, Basel, Switzerland) according to the manufacturer’s instructions. Relative gene expression levels were calculated using the 2^−ΔΔCt^ method and normalized to GAPDH expression. Each sample was analyzed in three technical replicates, and the mean Ct value of the three technical replicates was used for relative expression analysis. Primer sequences used in this study are listed in Supplementary Table S1.

### 2.7. Bulk RNA Sequencing and Analysis

For bulk RNA sequencing, independent differentiation runs were generated from one randomly selected iPSC clone per donor for clonal consistency. At the terminal stage of differentiation, osteogenic and chondrogenic cells were harvested, with five independent differentiation replicates per donor (from the same clone) for the osteogenic lineage and four for the chondrogenic lineage. Cells were enzymatically dissociated, collected by centrifugation, immediately snap-frozen in liquid nitrogen, and submitted to BGI Genomics for RNA sequencing. Sequencing libraries were generated and sequenced on the DNBSEQ platform using paired-end 150 bp reads (PE150). After quality control and removal of low-quality reads, clean reads were aligned to the human reference genome (GRCh38), and gene-level expression profiles were quantified.

Principal component analysis (PCA) was performed to assess the overall transcriptomic variation and sample clustering among groups. Differential gene expression analysis between TACS and CTRL samples was conducted using the DESeq2 package in R 4.5.1. Genes meeting the predefined significance criteria were considered differentially expressed genes. Kyoto Encyclopedia of Genes and Genomes (KEGG) pathway enrichment analysis was performed to characterize biological pathways associated with the differentially expressed genes. In addition, gene set enrichment analysis (GSEA) was conducted using ranked transcriptomic profiles to identify pathways showing coordinated expression changes between groups.

### 2.8. Prediction of TBX6-Binding Sites

Potential *TBX6*-binding sites within the promoter regions of mucin-type O-glycan biosynthesis-related genes were predicted using the JASPAR database. The consensus position frequency matrix for human *TBX6* was obtained from JASPAR, and the 2 kb genomic regions upstream of the transcription start sites (TSSs) of *GALNT3*, *GALNT5*, *GALNT7*, *GALNT10*, *GCNT1*, and *ST6GALNAC2* were screened for putative *TBX6*-binding motifs. Candidate binding sites were ranked according to their relative motif scores, and the three highest scoring sites for each gene were selected for visualization. The relative motif scores and genomic positions of the predicted sites were subsequently displayed as a heatmap and schematic promoter maps, respectively. These analyses were used to identify potential *TBX6*-responsive regulatory regions for subsequent experimental validation.

### 2.9. Western Blot Analysis

Total protein was extracted from iPSC-derived osteogenic cells using RIPA lysis buffer supplemented with protease inhibitors, and protein concentrations were determined using a BCA protein assay. Equal amounts of protein were separated by SDS-PAGE and transferred onto polyvinylidene difluoride membranes. After blocking, the membranes were incubated overnight at 4 °C with a mouse monoclonal anti-O-GalNAc/O-GlcNAc antibody (ARG22636, arigo Biolaboratories, Taiwan, China; 1:1000) or a rabbit monoclonal anti-GAPDH antibody (A19056, ABclonal, Wuhan, China). After washing, the membranes were incubated with the corresponding horseradish peroxidase (HRP)-conjugated secondary antibodies, including HRP-conjugated goat anti-mouse IgM (μ-chain specific; SA00012-6, Proteintech, Wuhan, China) for O-GalNAc/O-GlcNAc detection and HRP-conjugated secondary antibody (A0208, Beyotime, Shanghai, China) for GAPDH detection. Protein signals were visualized using an enhanced chemiluminescence (ECL) detection system. GAPDH was used as the loading control.

### 2.10. Statistical Analysis

Statistical analyses were performed using GraphPad Prism 10 and R 4.5.1. Data are presented as the mean ± standard deviation (SD) unless otherwise indicated. For qRT-PCR analyses, each sample was measured in three technical replicates; the technical replicates were averaged to obtain a single value per biological sample and were not treated as independent biological observations. Comparisons between the CTRL and TACS groups were performed using biological replicates (independent differentiation experiments) with an unpaired two-tailed Student’s *t*-test. Statistical significance is indicated in the figures as follows: * *p* < 0.05, ** *p* < 0.01, *** *p* < 0.001, and **** *p* < 0.0001. The number and type of replicates used in each experiment are specified in the corresponding figure legends.

## 3. Results

### 3.1. Clinical and Genetic Characterization of the Patient with TBX6-Associated Congenital Scoliosis

**Figure 1:**
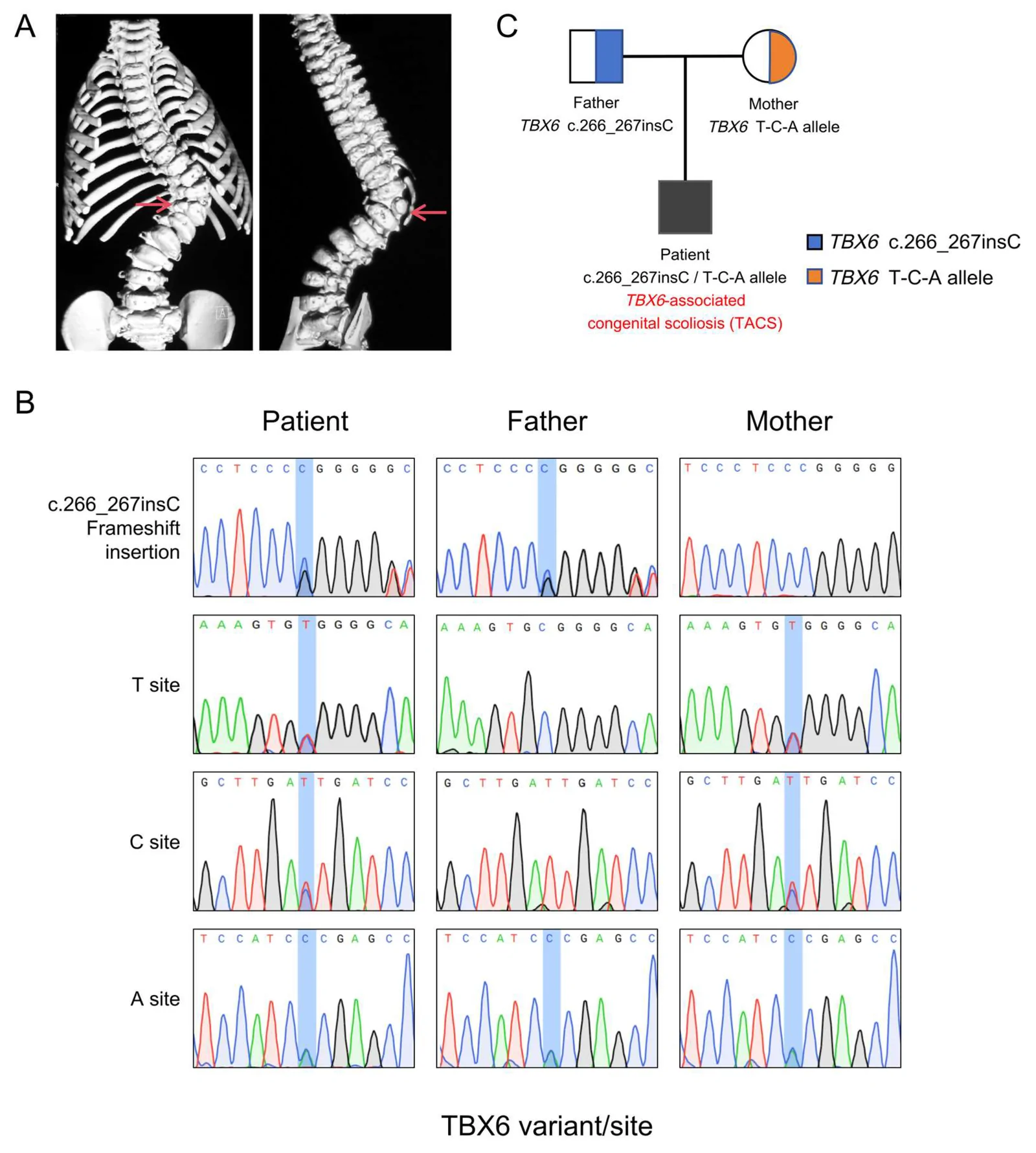
Clinical phenotype and compound inheritance of *TBX6*-associated congenital scoliosis. (**A**) Computed tomography (CT) images showing a congenital hemivertebra at the T12–L1 level, indicated by red arrows. (**B**) Sanger sequencing chromatograms of the patient and both parents showing the TBX6 frameshift variant (c.266_267insC) and the three SNPs constituting the hypomorphic T-C-A haplotype. Variant positions are highlighted in blue. (**C**) Schematic representation of the compound-inheritance pattern showing paternal inheritance of the *TBX6* frameshift variant and maternal inheritance of the hypomorphic T-C-A haplotype.

Clinical and genetic evaluation established a diagnosis of TACS in the proband. Computed tomography (CT) performed at the initial diagnosis revealed a hemivertebra at the T12–L1 level, confirming the presence of a congenital vertebral malformation (Figure 1A). The proband subsequently underwent corrective spinal surgery at 2 years of age. Sanger sequencing of the proband and both parents identified a heterozygous *TBX6* frameshift variant (c.266_267insC) in the proband and demonstrated its paternal origin. In parallel, Sanger analysis of the three single-nucleotide polymorphisms constituting the hypomorphic T-C-A haplotype confirmed that this risk haplotype was inherited from the unaffected mother. Representative Sanger chromatograms for c.266_267insC and the three SNP loci are shown in Figure 1B, with the variant positions highlighted in blue. These findings established that the *TBX6* frameshift variant and the hypomorphic T-C-A haplotype were present in trans in the proband, consistent with the established compound-inheritance model of TACS (Figure 1C). At a follow-up visit conducted in the 16th postoperative year, the proband showed a favorable clinical outcome without surgery-related complications and had an SRS-22r score of 4.6. PBMCs obtained from the proband and his unaffected mother were subsequently reprogrammed to establish patient-derived TACS and CTRL iPSC lines for downstream disease-modeling experiments.

### 3.2. Establishment and Validation of Patient-Derived iPSCs

**Figure 2:**
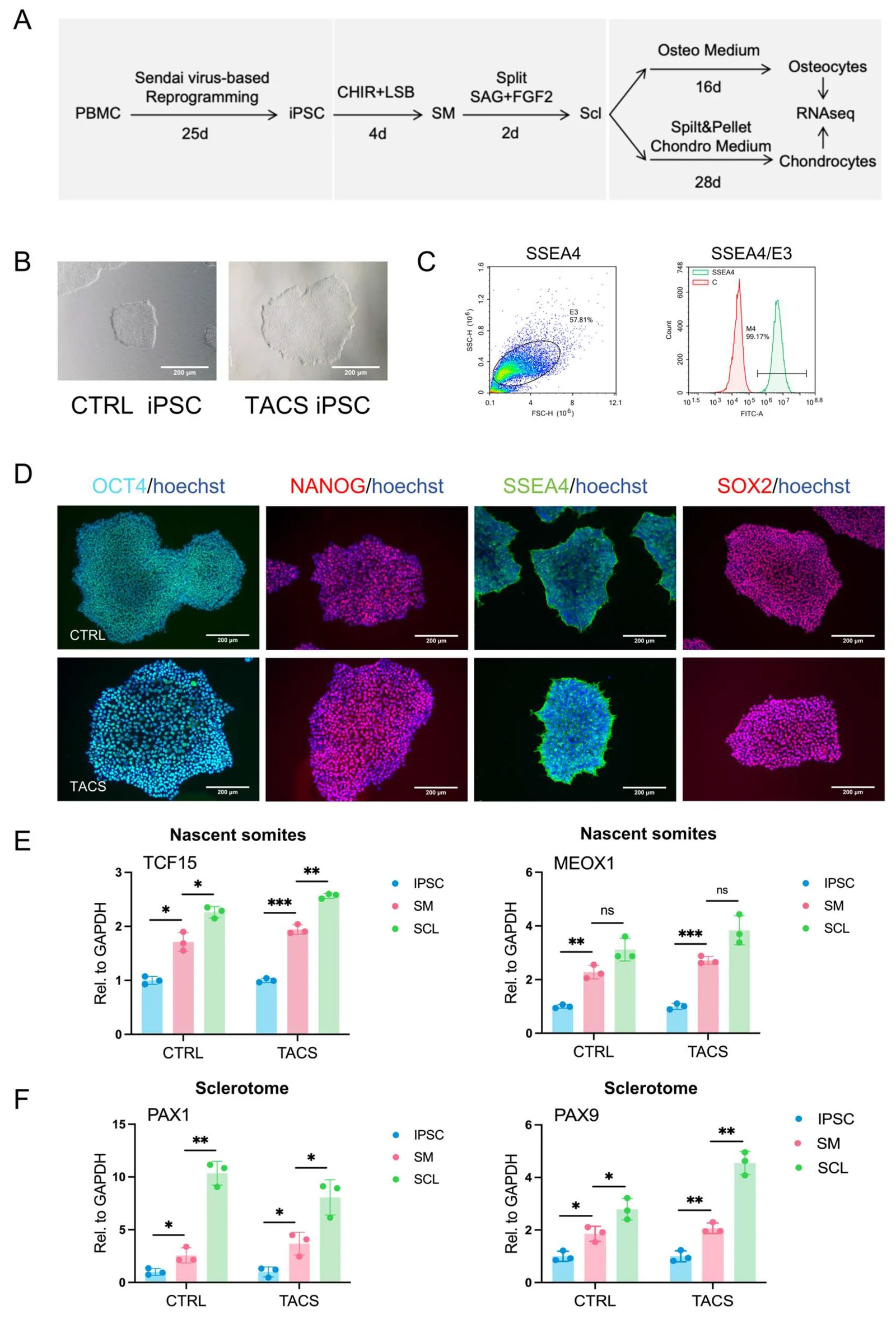
Establishment, characterization and developmental trajectory validation of patient-derived iPSCs. (**A**) Schematic diagram depicting iPSC generation and stepwise differentiation into paraxial mesoderm, nascent somites, sclerotome, and downstream osteogenic and chondrogenic lineages. (**B**) Representative bright-field images of iPSC colonies from CTRL and TACS. (**C**) Flow cytometry analysis showing SSEA4 expression in TACS iPSCs, with 99.17% SSEA4-positive cells. (**D**) Immunofluorescence staining of pluripotency markers SOX2, NANOG, SSEA4, and OCT4 in established iPSCs. Nuclei were counterstained with DAPI. (**E**,**F**) Expression of the Nascent somites (**E**) and Sclerotome (**F**) markers by qRT-PCR shown as average ± SEM (*n* = 3). *, *p* < 0.05; **, *p* < 0.01; ***, *p* < 0.001, ns, not significant. Data are shown as relative expression to GAPDH levels.

PBMCs obtained from the patient with TACS and his unaffected mother were reprogrammed using a Sendai virus-based system. After 25 days of reprogramming, colonies displaying typical human pluripotent stem cell morphology were selected and expanded to establish stable iPSC lines (Figure 2A,B). Flow cytometric analysis of the patient-derived TACS iPSCs showed that 99.17% of the analyzed cells expressed the pluripotency associated surface marker SSEA4 (Figure 2C). Immunofluorescence staining further revealed robust expression of *SOX2*, *NANOG*, *SSEA4*, and *OCT4*, consistent with maintenance of a pluripotent state (Figure 2D). Additional characterization showed normal karyotypes in both CTRL and TACS iPSC lines, indicating preserved chromosomal integrity following reprogramming and expansion (Supplementary Figure S1A,B). Both lines also exhibited strong alkaline phosphatase activity, further supporting their undifferentiated status (Supplementary Figure S1C,D). Sanger sequencing of the TACS iPSC line verified retention of the compound *TBX6* genotype after reprogramming, indicating stable preservation of the disease-associated genetic background in the patient-derived cells (Supplementary Figure S1E).

To mimic human vertebral development in vitro and assess the impact of *TBX6* dysfunction on osteogenic and chondrogenic differentiation, the iPSCs were subjected to a stepwise differentiation protocol adapted from Xi H et al. [11], progressing through paraxial mesoderm, nascent somite, and sclerotome stages before subsequent osteogenic or chondrogenic differentiation (Figure 2A). qRT-PCR confirmed induction of the somite markers *MEOX1* and *TCF15* and the sclerotome marker *PAX1* and *PAX9* during differentiation in both CTRL and TACS groups (Figure 2E,F). These results confirmed that both iPSC lines acquired the expected developmental identities and were suitable for subsequent comparative analyses of osteogenic and chondrogenic differentiation.

### 3.3. Impaired Osteogenic and Chondrogenic Differentiation in TACS

**Figure 3:**
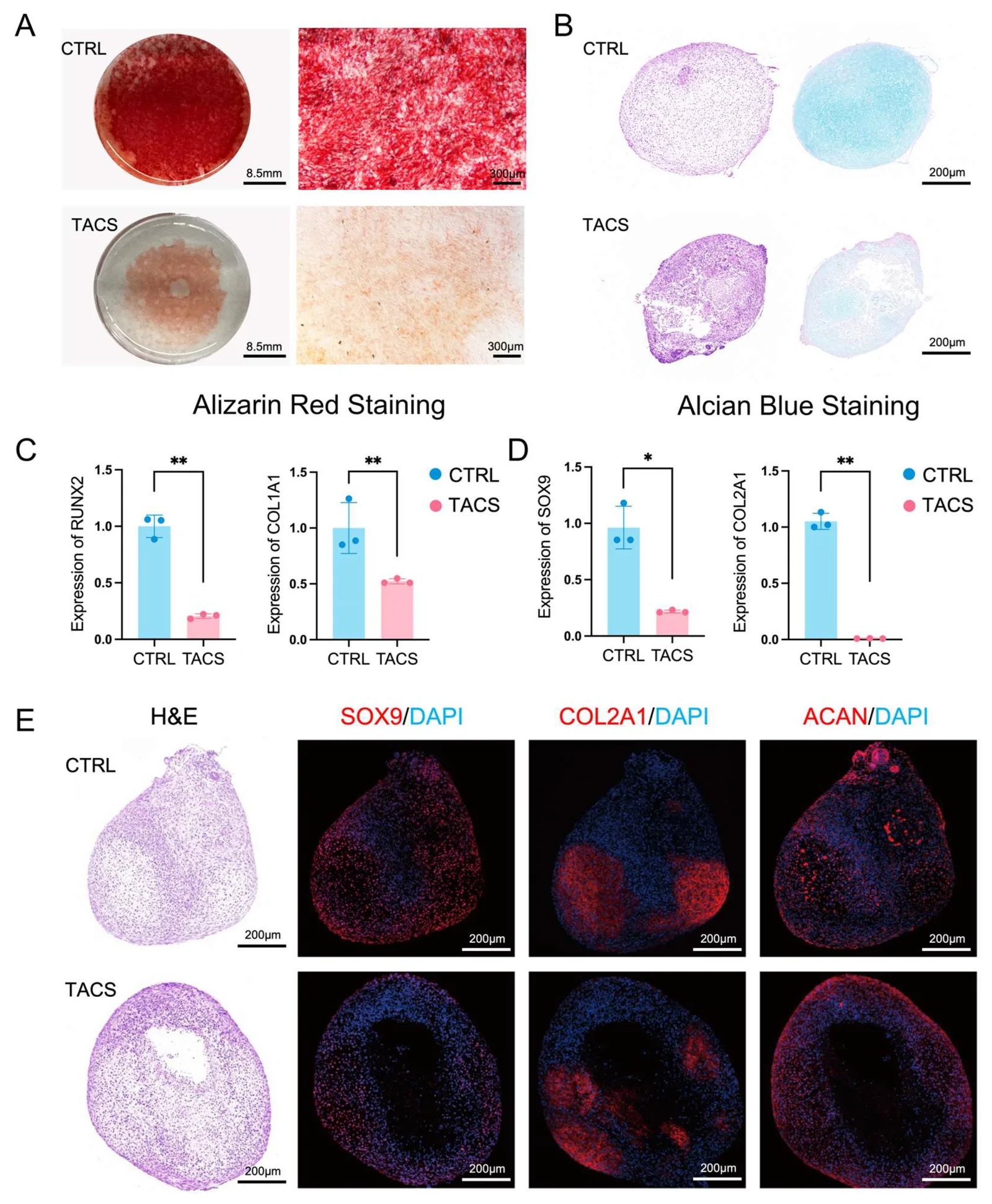
Defective Osteogenic and chondrogenic lineage differentiation of TACS patient-derived iPSCs. (**A**) Alizarin Red S staining assessing mineralized matrix deposition in CTRL and TACS iPSC-derived osteogenic cells. (**B**) Alcian Blue staining assessing cartilage matrix deposition in CTRL and TACS iPSC-derived chondrogenic pellets. (**C**) Expression of osteogenic markers *RUNX2* and *COL1A1* by qRT-PCR, shown as average ± SEM (*n* = 3 independent differentiation experiments). (**D**) Expression of chondrogenic markers *SOX9* and *COL2A1* by qRT-PCR, shown as average ± SEM (*n* = 3 independent differentiation experiments). (**E**) Histological and immunofluorescence characterization of CTRL and TACS iPSC-derived chondrogenic pellets by hematoxylin-eosin (H&E) staining and immunofluorescence staining for *SOX9*, *COL2A1*, and *ACAN*. Nuclei were counterstained with DAPI. *, *p* < 0.05; **, *p* < 0.01. Data are shown as relative expression to *GAPDH* levels.

Following lineage-directed differentiation, clear differences were observed between CTRL and TACS iPSC-derived osteogenic and chondrogenic cells. Alizarin Red S staining showed reduced mineralized matrix deposition in TACS-derived osteoblasts compared with CTRL group (Figure 3A). Consistent with this phenotype, qRT-PCR analysis showed significantly reduced expression of the osteogenic markers *RUNX2* and *COL1A1* in TACS-derived osteogenic cells (Figure 3C), further supporting attenuated osteogenic differentiation.

A similar attenuation was observed during chondrogenic differentiation. Alcian Blue staining showed reduced cartilage matrix deposition in TACS-derived chondrogenic pellets compared with CTRL pellets (Figure 3B). Consistently, qRT-PCR analysis demonstrated significantly reduced expression of the chondrogenic markers *SOX9* and *COL2A1* in the TACS group (Figure 3D). Histological examination by H&E staining revealed less prominent cartilage-like tissue organization, while immunofluorescence staining showed reduced expression of the chondrogenic marker *SOX9* and the cartilage matrix proteins *COL2A1* and *ACAN* in TACS-derived chondrogenic pellets (Figure 3E).

Together, these findings demonstrate attenuated osteogenic and chondrogenic differentiation in the TACS iPSC model.

### 3.4. Transcriptomic Profiles of Osteogenic and Chondrogenic Lineages

**Figure 4:**
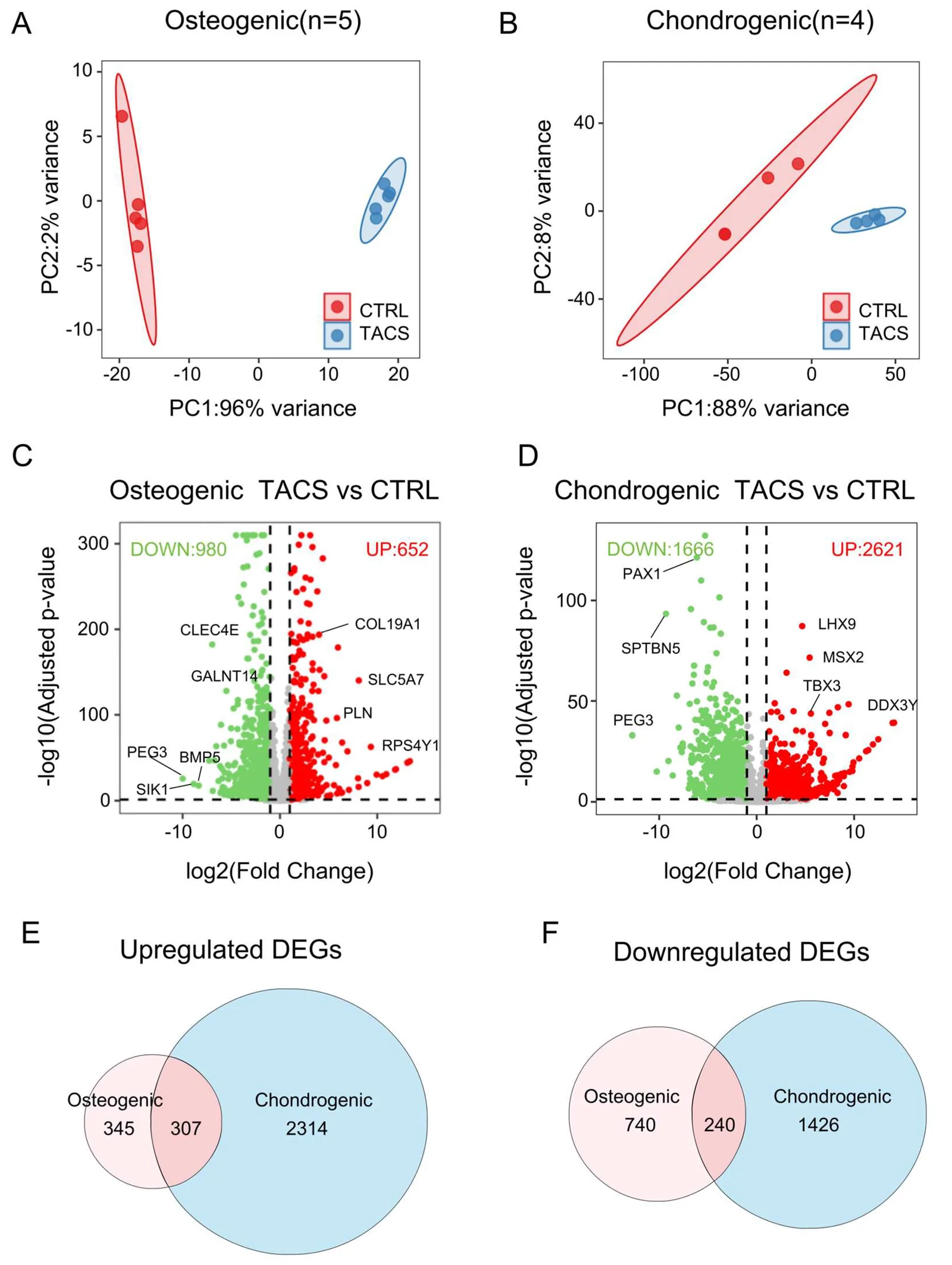
Transcriptomic profiling identifies unique and common molecular alterations in TACS−derived osteogenic and chondrogenic cells. (**A**) PCA plot illustrating transcriptomic profiles of CTRL and TACS iPSC−derived osteogenic cells. (**B**) PCA plot illustrating the transcriptomic separation between CTRL and TACS iPSC−derived chondrogenic cells. Two CTRL samples (rg_c2, rg_c3) exhibited highly similar expression profiles and closely overlapped on the plot. (**C**,**D**) Volcano plots illustrating differentially expressed genes (DEGs) between ctrl and TACS groups in osteogenic (**C**) and chondrogenic (**D**) cells. DEGs were defined by the threshold of |log2FC| ≥ 1 and adjusted *p* < 0.05. Grey dots represent genes with no significant differential expression, and dashed lines indicate the cut−off values for DEG screening. (**E**,**F**) Venn diagrams showing the overlap of significantly upregulated (**E**) and downregulated (**F**) DEGs between osteogenic and chondrogenic differentiation lineages.

To characterize the molecular alterations associated with TACS during osteogenic and chondrogenic differentiation, RNAseq was performed on CTRL and TACS iPSC-derived cells. Principal component analysis (PCA) revealed clear segregation of CTRL and TACS samples in both osteogenic and chondrogenic lineages, with biological replicates clustering within their respective groups, demonstrating robust and reproducible differences in the transcriptional landscape between CTRL and TACS cells during osteochondral differentiation. (Figure 4A,B).

Differential expression analysis using DESeq2 revealed extensive transcriptional remodeling in TACS-derived cells. At thresholds of |log2FC| ≥ 1 and adjusted *p* < 0.05, 652 genes were upregulated and 980 were downregulated in the osteogenic lineage, whereas 2621 genes were upregulated and 1666 were downregulated in the chondrogenic lineage (Figure 4C,D). The larger number of DEGs identified in chondrogenic cells indicates a broader transcriptional divergence between CTRL and TACS during chondrogenic differentiation.

Cross-lineage comparison identified 307 commonly upregulated and 240 commonly downregulated genes, together with substantial lineage specific transcriptional changes in both osteogenic and chondrogenic cells (Figure 4E,F). These shared differentially expressed genes were further subjected to functional enrichment analysis to identify molecular pathways commonly altered across the two lineages.

### 3.5. Dysregulation of Mucin-Type O-Glycan Biosynthesis in TACS

**Figure 5:**
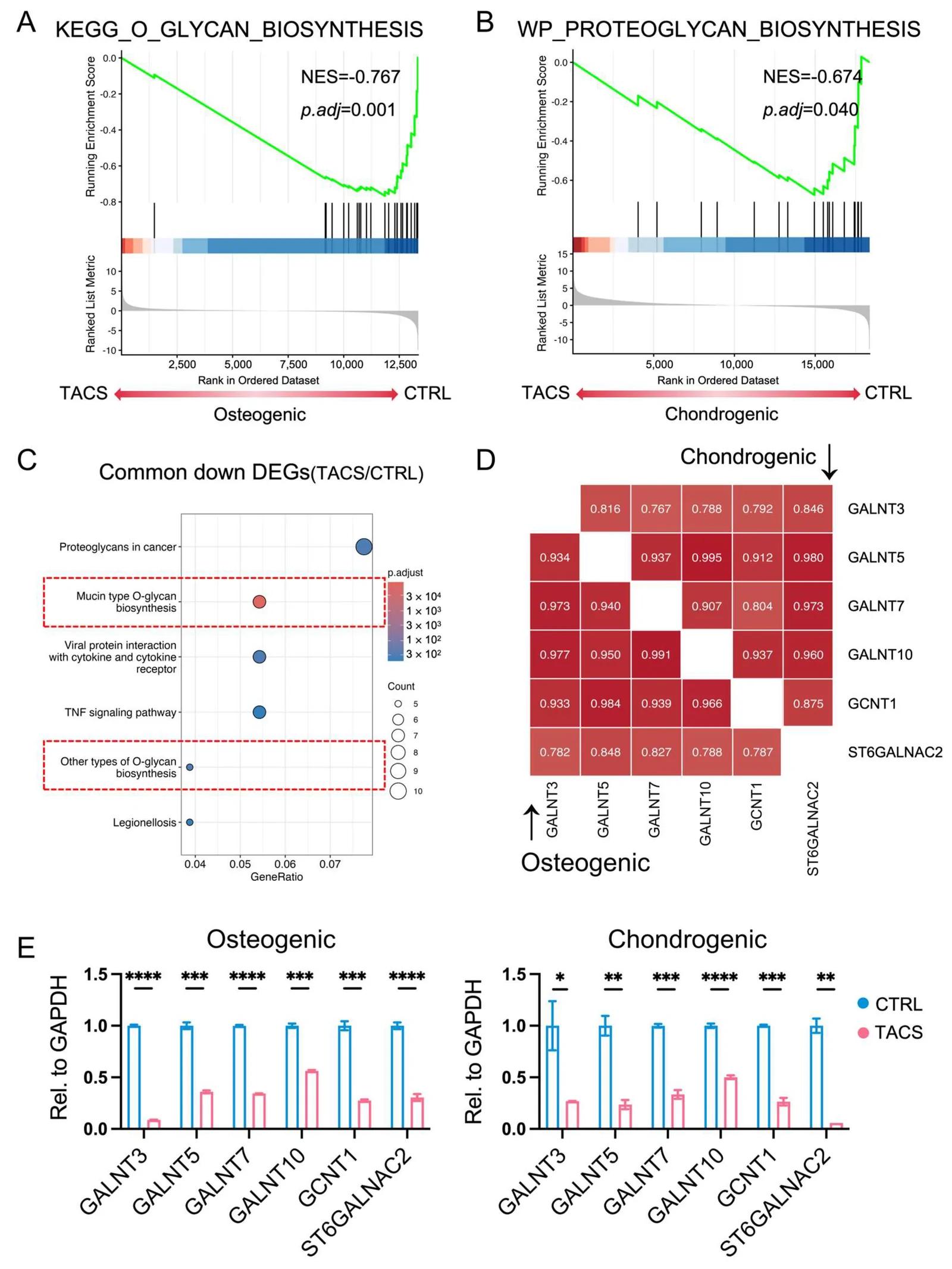
Suppressed expression of the mucin-type O−glycan biosynthesis-related transcriptional program. (**A**,**B**) Gene Set Enrichment Analysis (GSEA) showing enrichment of the mucin−type O−glycan biosynthesis gene set in CTRL versus TACS-derived osteogenic (**A**) and chondrogenic (**B**) cells. (**C**) KEGG pathway enrichment analysis of overlapping down−regulated genes shared by TACS osteogenic and chondrogenic lineages. (**D**) Pearson correlation analysis of expression levels among mucin−type O−glycan biosynthesis−related genes (*GALNT3*, *GALNT5*, *GALNT7*, *GALNT10*, *GCNT1*, and *ST6GALNAC2*). The lower-left triangle displays gene correlation coefficients from osteogenic cells, while the upper−right triangle shows correlations from chondrogenic cells. (**E**) Expression of mucin-type O-glycan biosynthesis related genes (*GALNT3*, *GALNT5*, *GALNT7*, *GALNT10*, *GCNT1* and *ST6GALNAC2*) validated by qRT−PCR in CTRL and TACS iPSC−derived osteogenic and chondrogenic cells. Data are shown as average ± SEM (*n* = 3 independent differentiation experiments). *, *p* < 0.05; **, *p* < 0.01; ***, *p* < 0.001; ****, *p* < 0.0001; ns, not significant. Expression levels were normalized to *GAPDH*.

To characterize the biological processes associated with the transcriptional alterations in TACS, KEGG enrichment analysis was first performed on the downregulated genes from the osteogenic and chondrogenic lineages. Osteogenic cells showed enrichment of pathways related to cell adhesion, proliferative regulation, and glycosylation, whereas chondrogenic cells were predominantly enriched for pathways associated with cell–matrix interactions and extracellular matrix signaling, including integrin signaling, focal adhesion, and ECM–receptor interaction (Supplementary Figure S2A,B). Notably, mucin-type O-glycan biosynthesis emerged as a commonly altered pathway in both lineages. Consistent with this observation, GSEA further demonstrated significant negative enrichment of the mucin-type O-glycan biosynthesis gene set in both osteogenic and chondrogenic TACS cells (Figure 5A,B). GSEA of the chondrogenic transcriptome additionally showed significant negative enrichment of the REAC_TOME_ECM_PROTE_OGLYCANS gene set in TACS cells (Supplementary Figure S2C), indicating coordinated suppression of an extracellular matrix-associated transcriptional program.

Because mucin-type O-glycan biosynthesis was consistently negatively enriched in both osteogenic and chondrogenic lineages, we next examined whether this shared pathway signal was associated with a common set of transcriptional changes. KEGG enrichment analysis of genes downregulated in both lineages again identified mucin-type O-glycan biosynthesis as a significantly enriched pathway (Figure 5C). Six genes involved in this pathway—*GALNT3*, *GALNT5*, *GALNT7*, *GALNT10*, *GCNT1*, and *ST6GALNAC2*—were commonly downregulated in both lineages. Pearson correlation analysis further revealed strong positive correlations among the expression levels of these genes in both osteogenic and chondrogenic cells, with pairwise correlation coefficients predominantly ranging from approximately 0.75 to 1.0 (Figure 5D). This coordinated expression pattern supports a pathway level transcriptional alteration rather than isolated changes in individual O-glycosylation-related genes. Consistent with the RNA-seq results, qRT-PCR analysis confirmed significantly reduced expression of all six genes in TACS-derived osteogenic and chondrogenic cells (Figure 5E). These findings support coordinated dysregulation of the mucin-type O-glycan biosynthesis-related transcriptional program in TACS.

**Figure 6:**
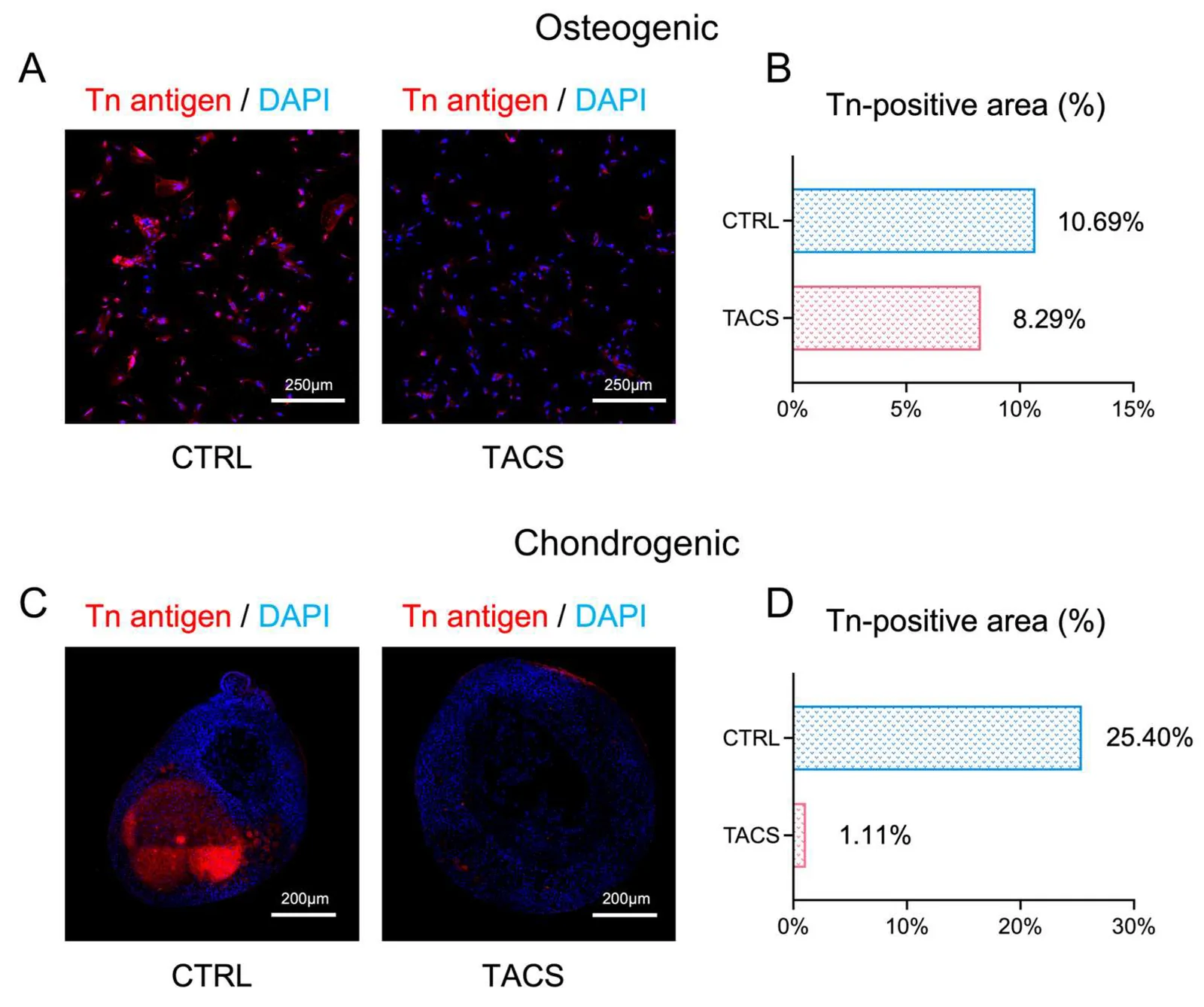
Reduced Tn-associated O-GalNAc signals in TACS-derived osteogenic and chondrogenic cells. (**A**,**C**) Representative immunofluorescence images of Tn antigen staining in CTRL and TACS-derived osteogenic (**A**) and chondrogenic (**C**) cells. Nuclei were counterstained with DAPI. (**B**,**D**) Percentage of Tn-positive area in the representative osteogenic (**B**) and chondrogenic (**D**) samples shown.

To determine whether these transcriptional alterations were accompanied by changes at the glycan level, we next performed immunofluorescence staining for the Tn antigen, an O-GalNAc-associated glycan epitope. TACS-derived osteogenic cells showed reduced Tn-antigen staining compared with CTRL cells, with the Tn-positive area decreasing from 10.69% to 8.29% (Figure 6A,B). A more pronounced reduction was observed in chondrogenic cells, with the Tn-positive area decreasing from 25.40% in CTRL cells to 1.11% in TACS cells (Figure 6C,D). Additionally, Western blot analysis using an anti-O-GalNAc/O-GlcNAc monoclonal antibody showed reduced O-GalNAc/O-GlcNAc-associated immunoreactivity in TACS-derived osteogenic cells compared with CTRL cells (Figure S3A). Together, these observations indicate that the transcriptional alterations in the mucin-type O-glycan biosynthesis program are accompanied by reduced Tn-associated O-GalNAc signals in TACS-derived cells.

### 3.6. Predicted TBX6-Binding Sites in O-Glycosylation-Related Genes

**Figure 7:**
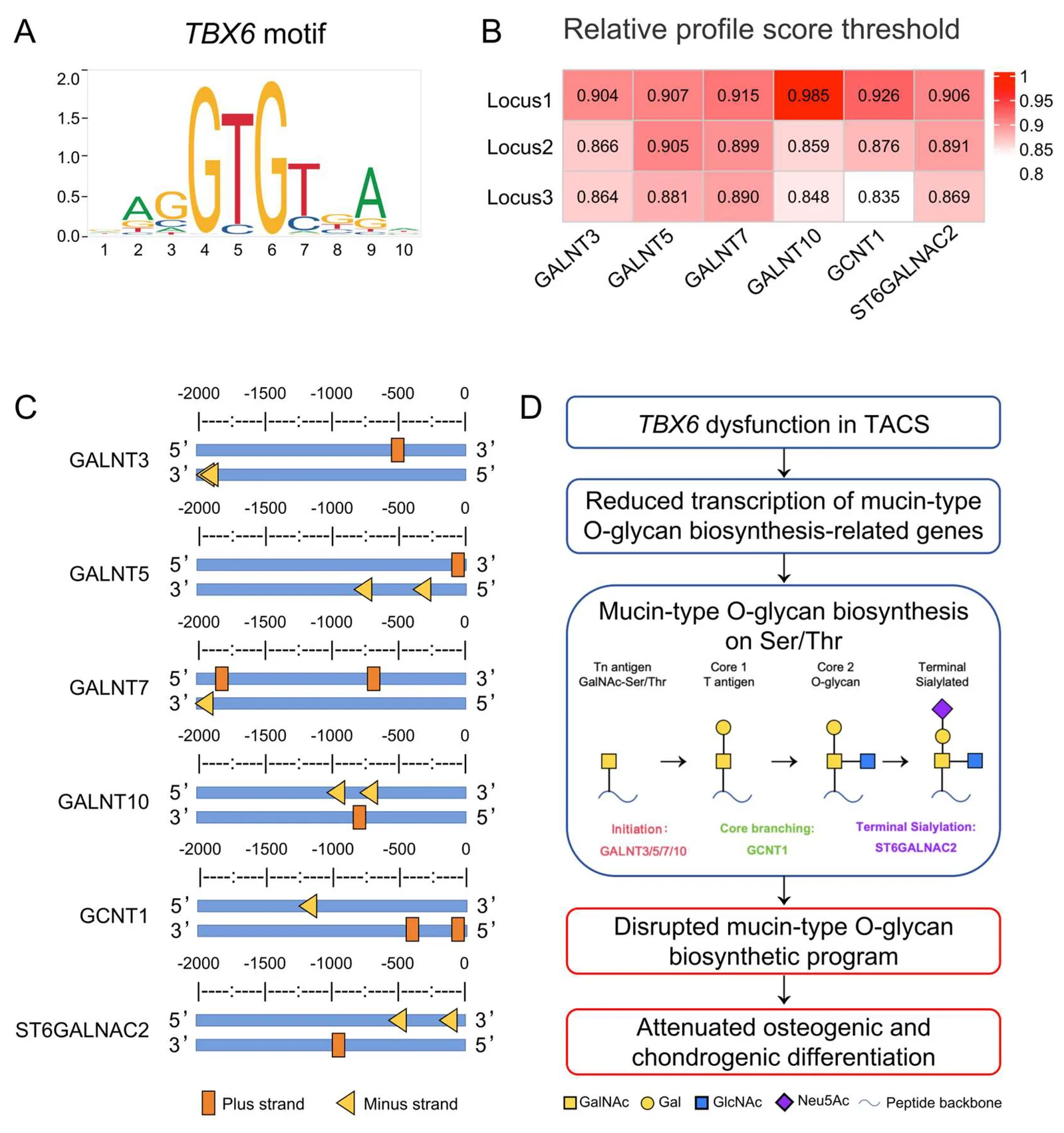
In silico prediction of putative *TBX6*−binding sites in mucin−type O−glycan biosynthesis−related genes. (**A**) Consensus DNA−binding motif of TBX6 obtained from the JASPAR database. (**B**) Heatmap illustrating relative motif scores of the top-three predicted TBX6−binding sites located within the 2 kb upstream promoter regions of *GALNT3*, *GALNT5*, *GALNT7*, *GALNT10*, *GCNT1* and *ST6GALNAC2*. (**C**) Schematic representation showing the genomic distribution of the top-three predicted *TBX6*−binding sites within the 2 −kb upstream regions of these genes. Positions are indicated relative to the transcription start site (TSS). (**D**) Proposed workingmodel schematic illustrating the mucin−type O−glycan biosynthetic cascade downstream of *TBX6* dysfunction. This schematic represents a hypothesis derived from the present data and does not demonstrate direct TBX6 binding or transcriptional regulation.

Given the consistent reduction in several mucin-type O-glycan biosynthesis related genes in TACS-derived osteogenic and chondrogenic cells, we performed an exploratory in silico analysis to determine whether the upstream regions of these genes contain sequences matching the consensus TBX6-binding motif. The consensus *TBX6*-binding motif was retrieved from the JASPAR database and used to scan the 2 kb regions upstream of the transcription start sites of *GALNT3*, *GALNT5*, *GALNT7*, *GALNT10*, *GCNT1*, and *ST6GALNAC2* (Figure 7A).

Candidate motif matches were identified within the upstream regions of all six genes. The three highest-scoring predicted sites for each gene showed similarity to the JASPAR TBX6 consensus motif, with relative profile scores ranging from approximately 0.84 to 0.99 (Figure 7B). Positional mapping further revealed that these candidate sites were distributed across distinct locations within the 2 kb upstream regions (Figure 7C). In conjunction with the expression changes observed across both lineages, these in silico results raise the possibility of a link between reduced *TBX6* activity and transcriptional perturbation of the mucin-type O-glycan biosynthetic machinery, although they do not establish direct TBX6 binding or regulation.

To integrate the phenotypic, transcriptomic, and motif-prediction results, we generated a working model schematic summarizing a potential sequence in which *TBX6* dysfunction is accompanied by reduced expression of key O-glycan biosynthesis related genes, with subsequent effects on downstream cellular processes relevant to osteogenic and chondrogenic differentiation (Figure 7D). This model represents a mechanistic hypothesis derived from the present data rather than direct evidence of TBX6-mediated transcriptional control. Direct TBX6 binding and transcriptional regulation will require experimental validation using chromatin-occupancy and functional regulatory assays.

## 4. Discussion

In this study, we generated a patient derived iPSC model for TACS and employed a step wise developmental differentiation platform to investigate the downstream impacts of *TBX6* dysfunction beyond early stage somitogenesis. TACS-derived cells exhibited attenuated osteogenic and chondrogenic differentiation, accompanied by convergent transcriptional alterations across the two skeletal lineages. Notably, mucin-type O-glycan biosynthesis was identified as a commonly dysregulated pathway, with consistent downregulation of *GALNT3*, *GALNT5*, *GALNT7*, *GALNT10*, *GCNT1* and *ST6GALNAC2* in both osteogenic and chondrogenic cell populations. Strong positive correlations observed between these genes further support coordinated, pathway wide transcriptional dysregulation, rather than independent, gene specific alterations. Consistent with these transcriptional findings, reduced Tn-associated O-GalNAc signals were observed in TACS-derived osteogenic and chondrogenic cells. Furthermore, the identification of predicted *TBX6* binding motifs in the upstream regulatory regions of these genes provides candidate regulatory regions for future investigation, although these in silico predictions do not establish direct TBX6 binding or transcriptional regulation. Collectively, our findings suggest that the molecular and differentiation alterations associated with TACS remain evident beyond the early developmental stages in which TBX6 has been most extensively characterized.

Previous genetic and experimental studies have established *TBX6* as a dosage sensitive regulator of vertebral development [8,9,10,11,12,18,19,20]. The characteristic combination of one loss of function allele and one hypomorphic allele has been functionally linked to congenital vertebral malformations in both humans and mice [8,18], whereas increased *TBX6* dosage can likewise disrupt vertebral patterning. These findings emphasize that tightly controlled *TBX6* activity is indispensable during axial skeleton development. Mechanistically, however, most prior work has focused on early stage developmental events, particularly paraxial mesoderm specification and somite segmentation [2,3,4,5,6,7,14,15,17,21,22], including the temporal regulation of Tbx6 during mesodermal lineage allocation and Ripply-dependent establishment of somite boundaries [4,5,6]. Our findings extend this conceptual framework by showing impaired osteogenic and chondrogenic differentiation in TACS-derived cells after cells have progressed through somite and sclerotome like developmental stages. This observation suggests that the developmental consequences associated with altered TBX6 activity may extend beyond somite formation and remain evident during later stages of vertebral skeletal lineage development.

The concurrent impairment of osteogenic and chondrogenic differentiation holds clear developmental relevance to the vertebral phenotype observed in TACS. Vertebral formation is not an exclusively osteogenic process; rather, somite derived sclerotomal cells undergo cellular condensation and chondrogenic differentiation before much of the vertebral skeleton forms via endochondral ossification [1,15,16,23]. Accordingly, cartilage formation and bone maturation represent interconnected, sequential stages of axial skeletal development [1,23]. In this context, the parallel reductions in mineralization, cartilage matrix deposition, and lineage specific marker expression detected in TACS derived cells are more consistent with broad ranging disturbance of osteochondral development than with an isolated defect within either single lineage. This dual lineage phenotype therefore provides a developmental rationale for the vertebral malformations characteristic of TACS, and suggests that disrupted coordination of osteochondral maturation may contribute to structural defects of the vertebral column.

The shared dysregulation of mucin type O glycan biosynthesis provides a potential molecular entry point to explain this later onset developmental phenotype. Among the genes identified in our dataset, *GALNT3* has the best established link to skeletal biology. In developing cartilage, *Galnt3* is transcriptionally responsive to *Runx2*, and genetic perturbation of *Galnt3* alters chondrocyte maturation and endochondral ossification [24]. Notably, both loss of function and excessive *Galnt3* expression can disturb skeletal development: deficiency delays embryonic endochondral ossification, whereas chondrocyte specific overexpression disrupts growth plate architecture and impairs deposition of aggrecan and glycosaminoglycan rich extracellular matrix [24]. More recently, *Runx2* was shown to directly regulate *Galnt3* transcription in osteoblast lineage cells, while *Galnt3* modulates osteoid mineralization through stabilization of *Fgf23* [25]. These published observations are consistent with the parallel reductions in osteogenic differentiation and O-glycosylation related gene expression seen in our model, although they do not define the direction of causal relationships within the TACS context. Importantly, the biological significance of our results is unlikely to be explained by *GALNT3* alone. GalNAc transferases exhibit overlapping yet non redundant substrate specificities, and loss of individual GALNT isoenzymes can drive distinct biological outcomes, including altered cell differentiation and cell–matrix adhesion [26,27]. The coordinated transcriptional changes affecting multiple GALNT family enzymes together with downstream glycosyltransferases therefore point toward disruption of a broader O-GalNAc associated transcriptional program, rather than isolated dysfunction of a single glycosyltransferase. Consistent with these transcriptional alterations, Tn-antigen immunofluorescence showed reduced Tn-associated O-GalNAc signals in TACS-derived osteogenic and chondrogenic cells. Western blot analysis of osteogenic cells using an anti-O-GalNAc/O-GlcNAc monoclonal antibody provided additional supportive evidence of altered glycosylation-associated immunoreactivity. Together, these findings indicate that the transcriptional alterations are accompanied by changes in glycan-associated signals, although the current assays do not define the precise composition, structure, or site-specific alterations of the affected O-glycans.

The chondrogenic transcriptome provides additional evidence that this glycosylation associated alteration occurs within a substantially remodeled extracellular environment. In addition to O-linked glycosylation of mucins, which was negatively enriched and contained *GALNT3*, *GALNT5*, *GALNT7*, *GALNT10*, *GCNT1*, and *ST6GALNAC2* within its leading edge genes, TACS-derived chondrogenic cells showed strong negative enrichment of extracellular matrix organization, ECM proteoglycans, glycosamino glycan metabolism, and the NABA proteoglycan gene set. The leading edge subsets of these pathways contained structural and matrix organizing genes including *HSPG2*, *BGN*, *DCN*, *LUM*, *HAPLN1*, *ACAN*, *COL2A1*, and *COMP*. Importantly, mucin-type O-GalNAc glycans and proteoglycan glycosaminoglycan chains represent biochemically distinct forms of glycosylation [27,28]; therefore, their parallel alteration should not be interpreted as evidence that one pathway directly produces the other. Rather, the convergence of these independent glycan and matrix programs suggests broader disruption of extracellular matrix maturation in TACS-derived cartilage. This interpretation is consistent with the known capacity of O-GalNAc glycosylation to influence protein processing, trafficking, receptor function, and cell adhesion [27]. Experimental studies have further demonstrated that O-glycosyltransferase activity can control the secretion and localization of extracellular-matrix ligands and thereby modulate integrin-dependent cell adhesion during development.

A potential signaling bridge connecting this remodeled extracellular environment to the impaired chondrogenic phenotype is indicated by coordinated transcriptional changes affecting cell–matrix and intracellular signaling pathways. Integrin mediated cell surface interactions were negatively enriched in TACS derived chondrogenic cells; leading edge genes included integrin subunits *ITGB1*, *ITGB5*, *ITGA5*, *ITGA10*, and *ITGA11*, alongside matrix related transcripts *COL2A1*, *FN1*, and *COMP*. Several of these identical integrin and matrix components also appeared within the negatively enriched *PI3K-AKT* gene set, revealing transcriptional convergence between extracellular matrix sensing and downstream intracellular signaling. While these transcript level data do not confirm reduced AKT kinase activity, the observation is biologically meaningful, since PI3K-AKT signaling is known to support chondrogenic differentiation in human mesenchymal stromal cells and facilitates TGF-β dependent SMAD2 phosphorylation and SOX9 protein accumulation [29]. Consistent with this functional connection, TGF-β signaling was also negatively enriched in TACS derived chondrogenic cells, with *TGFBR1*, *TGFB1*, *SMAD6*, *SMAD7*, and *BMP2* detected among its leading edge genes. Taken together, these results support a working model whereby altered glycosylation and matrix remodeling coincide with disruption of the ECM integrin dependent signaling niche, generating an environment less permissive for PI3K-AKT and TGF-β driven chondrogenic transcriptional programs. This conceptual framework offers a plausible biological explanation for the reduced *SOX9*, *COL2A1*, *ACAN* expression and impaired cartilage matrix formation observed in TACS derived cells, although the causal direction and hierarchical relationships among these molecular events remain to be experimentally defined.

Additional transcriptional signaling alterations indicate that the molecular phenotype extends beyond this ECM centered mechanistic axis. Specifically, TNF-α/NF-κB associated and mTORC1 associated gene sets were also strongly negatively enriched in the chondrogenic transcriptome. Nevertheless, both NF-κB and mTOR signaling exert stage and context dependent functions during cartilage development [30,31,32,33,34,35,36,37], limiting our ability to infer their functional activity or phenotypic contribution solely from transcriptional enrichment data. We therefore interpret these pathway changes as markers of broader signaling network remodeling, rather than core components of the primary mechanistic model proposed herein. This distinction is critical, as GSEA detects coordinated shifts in pathway level gene expression but cannot directly measure kinase activation, protein phosphorylation status, or real time signaling flux [38].

The upstream relationship between *TBX6* dysfunction and the O-glycosylation associated signature also remains to be established. Our motif analysis identified candidate *TBX6*-binding sequences within the 2 kb upstream regions of *GALNT3*, *GALNT5*, *GALNT7*, *GALNT10*, *GCNT1*, and *ST6GALNAC2*. The presence of predicted sites across multiple components of the pathway, together with their concordant expression changes in both skeletal lineages, raises the possibility that altered *TBX6* activity contributes to a broader glycosylation associated transcriptional program. Nevertheless, motif occurrence is not equivalent to transcription-factor occupancy and cannot establish direct regulation. Demonstrating such a relationship will require chromatin-based assays such as ChIP-qPCR, CUT&Tag, or CUT&RUN [39,40], complemented by promoter reporter experiments. In addition, restoration of *TBX6* activity in TACS cells, ideally using an isogenic gene-corrected line, would be necessary to determine whether normalization of the O-glycosylation-related program accompanies rescue of the osteogenic and chondrogenic phenotypes.

Several limitations should therefore be considered. First, the present study was based on a single TACS family, which limits the generalizability of the observed molecular and differentiation phenotypes. The maternal line was used as an asymptomatic familial comparator rather than a genotype-neutral control. The mother carries the hypomorphic T-C-A haplotype but not the patient’s TBX6 loss-of-function allele. Importantly, the T-C-A haplotype is a common hypomorphic TBX6 haplotype and is generally insufficient to cause TACS on its own; current evidence instead supports a dosage-dependent compound-inheritance model in which the T-C-A haplotype substantially increases disease risk when it occurs in trans with a rare TBX6 loss-of-function allele [8,18]. Thus, the within-family design provides a genetically informative comparison between an asymptomatic T-C-A-carrying background and the patient’s compound TBX6 loss-of-function/T-C-A genotype. Because the mother also shares part of the patient’s genetic background, this comparison reduces some of the genetic heterogeneity that would be introduced by a completely unrelated control. Nevertheless, the maternal line is neither genotype-neutral nor isogenic, and donor- and genetic-background-related effects cannot be excluded. Accordingly, the findings of the present study should be interpreted within the context of this patient–mother cell-line pair. Additional independent TACS families and, importantly, isogenic TBX6-corrected or rescue models will be required to determine the reproducibility and TBX6 specificity of the observed phenotypes.

Second, although Tn-antigen immunofluorescence and O-GalNAc/O-GlcNAc immunoblotting provide additional glycan-associated evidence beyond the transcriptional findings, these assays do not define the composition, structure, site occupancy, or glycoprotein substrates of the affected O-glycans. Glycomic and O-glycoproteomic approaches will therefore be required to characterize these alterations more precisely. Third, the proposed ECM–integrin–PI3K/AKT–TGF-β connection is based on transcriptomic convergence and prior biological evidence rather than direct signaling measurements. Analysis of integrin/FAK activity, AKT phosphorylation, and TGF-β/SMAD signaling would therefore provide important mechanistic validation. Finally, direct TBX6 occupancy and functional rescue remain to be established. Within these limitations, our findings support a model in which TACS-associated TBX6 dysfunction is accompanied by coordinated alteration of an O-glycosylation-related transcriptional program, together with broader remodeling of extracellular matrix and cell–matrix signaling programs and attenuated osteogenic and chondrogenic differentiation. These observations provide a potential molecular framework for investigating the relationships among altered TBX6 function, O-glycan-associated changes, and later disturbances in human vertebral osteochondral development.

## 5. Conclusions

In conclusion, our patient-derived iPSC model of TACS reveals that TACS-derived cells exhibit attenuated osteogenic and chondrogenic differentiation together with coordinated dysregulation of a mucin-type O-glycan biosynthesis-related transcriptional program and altered glycan-associated signals. These changes are accompanied by broader alterations in extracellular matrix, proteoglycan, and cell–matrix signaling pathways, suggesting a potential association between altered glycosylation-related programs and impaired vertebral osteochondral development. Although the direct regulatory relationship between TBX6 and these downstream molecular alterations remains to be established, our findings identify O-glycan-associated dysregulation as a molecular feature of this family-derived TACS model and provide a basis for further investigation using additional TACS lines and isogenic TBX6-corrected models.

## Data Availability

Raw sequencing data generated in this study have been deposited in the NCBI Sequence Read Archive (SRA) under BioProject accession number PRJNA1534602.

## Supplementary Materials

The following supporting information can be downloaded at https://www.mdpi.com/article/10.3390/cells15191785/s1: Figure S1:Characterization of patient-derived CTRL and TACS iPSC lines; Figure S2: Pathway enrichment analyses; Figure S3: O-GalNAc/O-GlcNAc immunoreactivity in osteogenic cells; Table S1: Primers used for gene expression analysis.

## Abbreviations

The following abbreviations are used in this manuscript:

TACS	*TBX6*-associated congenital scoliosis
PBMC	Peripheral blood mononuclear cell
CT	Computed tomography
iPSC	induced pluripotent stem cell
CTRL	maternal familial comparator
CS	Congenital scoliosis
GSEA	Gene Set Enrichment Analysis
SRS-22r	Scoliosis Research Society-22 revised questionnaire
SSEA4	Stage-specific embryonic antigen-4
AP	Alkaline phosphatase
IF	Immunofluorescence
DAPI	4′,6-Diamidino-2-phenylindole
H&E	Hematoxylin and eosin
PCR	Polymerase Chain Reaction
qRT-PCR	Quantitative reverse transcription polymerase chain reaction
RNA-seq	RNA sequencing
PCA	Principal component analysis
DEG	Differentially expressed gene
GO	Gene Ontology
KEGG	Kyoto Encyclopedia of Genes and Genomes
ECM	Extracellular matrix
TSS	Transcription start site
O-GALNAc	O-linked N-acetylgalactosamine
GalNAc	N-Acetylgalactosamine
GAG	Glycosaminoglycan
FGF	Fibroblast growth factor
PI3K	Phosphoinositide 3-kinase
AKT	Protein kinase B
TGF-β	Transforming growth factor beta
TNF	Tumor necrosis factor
NF-κB	Nuclear factor kappa B
mTORC1	Mechanistic target of rapamycin complex 1
ChIP	Chromatin immunoprecipitation
CUT&Tag	Chromatin immunoprecipitation followed by quantitative PCR
CUT&RUN	Cleavage under targets and tagmentation
FDR	False discovery rate
NES	Normalized enrichment score
WB	Western blot
