# Supplementary material for: Patient-Derived iPSC Modeling Reveals Dysregulation of the Mucin-Type O-Glycan Biosynthesis-Related Transcriptional Program in TBX6-Associated Congenital Scoliosis
Source: Cells. 2026 Sep 30;15(19):1785. doi: 10.3390/cells15191785 (PMC13664113; doi:10.3390/cells15191785)
Supplement: Supplementary file 1 [file cells-15-01785-s001.zip › cells-4577565-supplementary.pdf]

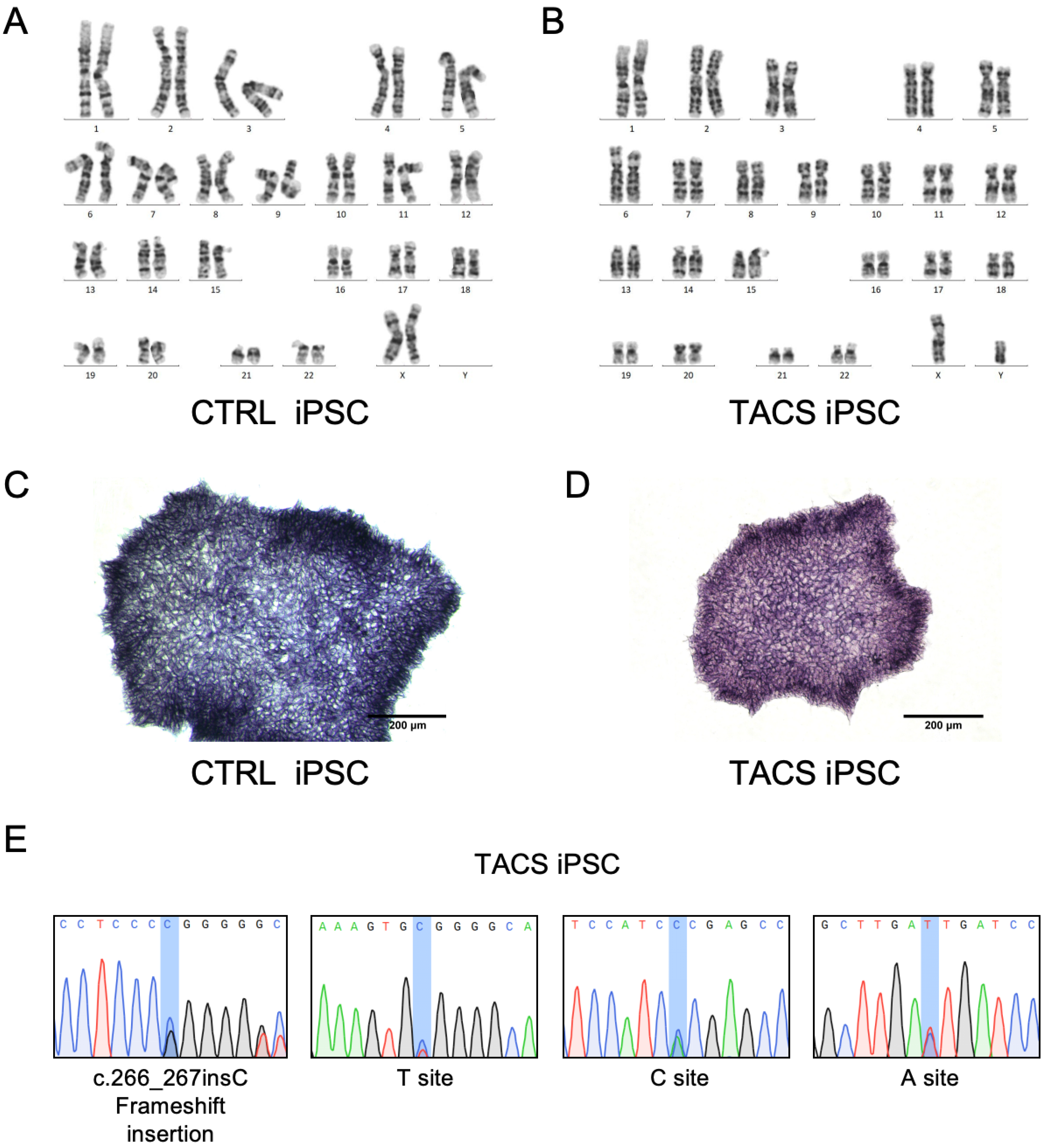

Supplementary Figure S1 Characterization of patient-derived CTRL and TACS iPSC lines. (A-B) Chromosomal karyotype analysis showing a normal karyotype in CTRL and TACS iPSCs. (C-D) Alkaline phosphatase staining confirming the undifferentiated pluripotent status of iPSC colonies. (E) Sanger-sequencing chromatogram verifying the compound *TBX6* genotype in TACS-derived iPSCs.

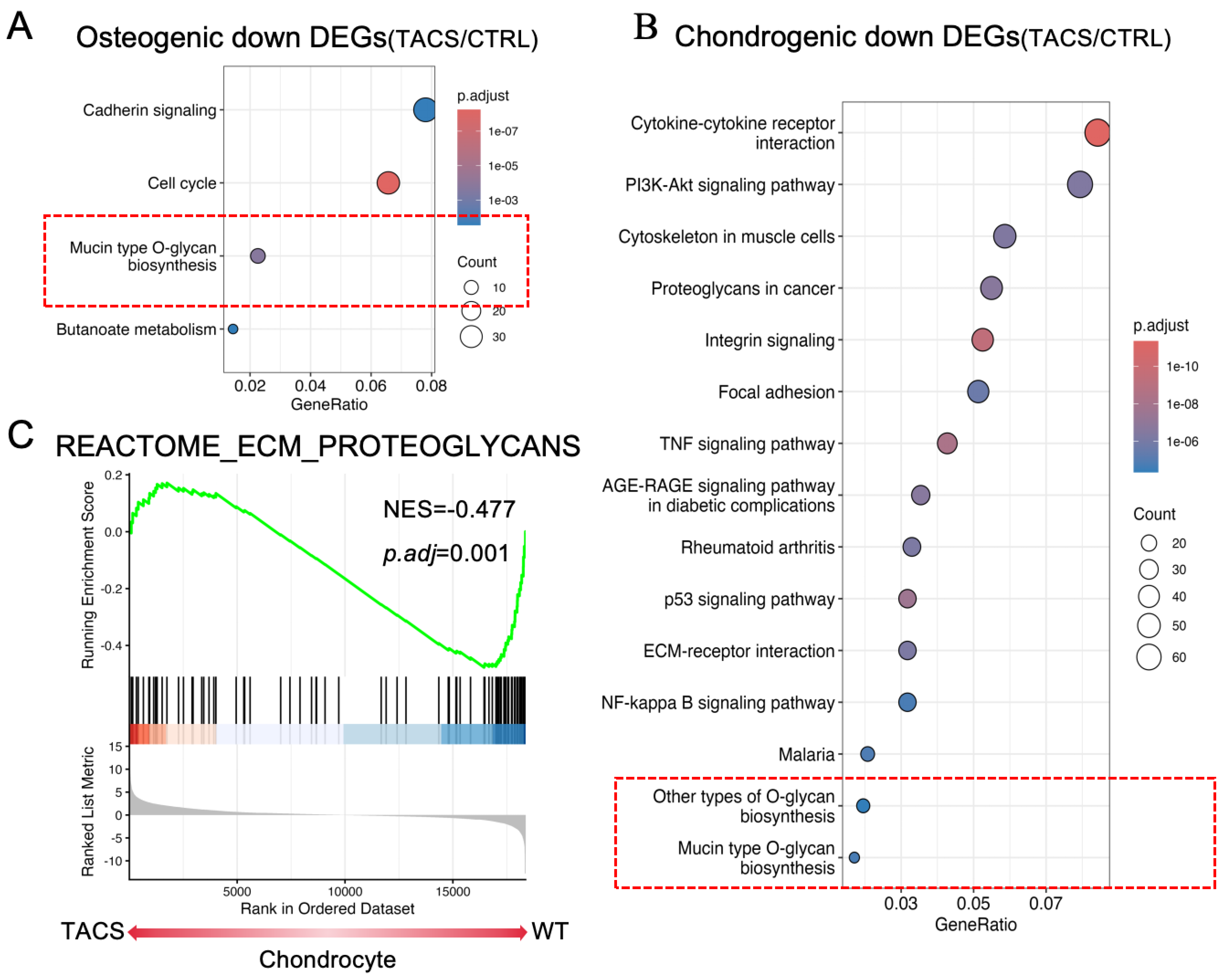

Supplementary Figure S2 Pathway enrichment analyses. (A) KEGG enrichment of down-regulated genes in osteogenic cells. (B) KEGG enrichment of down-regulated genes in chondrogenic cells. (C) GSEA plot showing enrichment of the REACTOME_ECM_PROTEOGLYCANS pathway using the complete ranked transcriptome of chondrogenic cells.

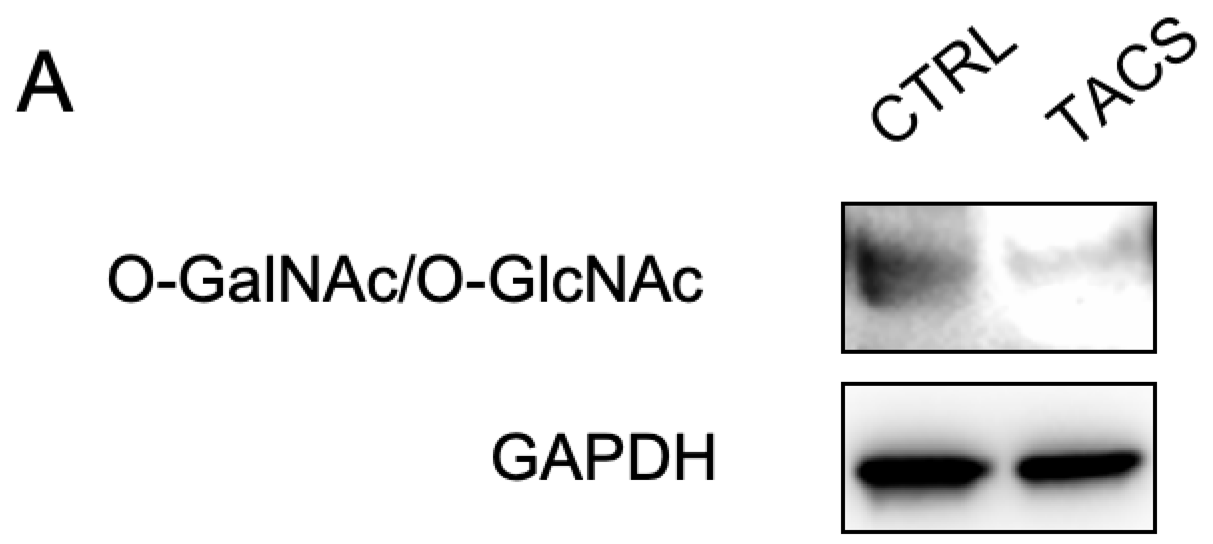

Supplementary Figure S3 O-GalNAc/O-GlcNAc immunoreactivity in osteogenic cells. (A) Western blot analysis of CTRL and TACS iPSC-derived osteogenic cells using an anti-O-GalNAc/O-GlcNAc monoclonal antibody. GAPDH served as the loading control.

Supplementary Table S1 Primers used for gene expression analysis

Gene name	Forward primer	Reverse primer
PAX1	TCGCTATGGAGCAGACGTATG	GCTGCCGACTGATGTCACA
PAX9	AGCAGGGTCATTACGACTCAT	CTGGGGTACGAGTAGATGTGG
MEOX1	GGCAGCGTACCCTGACTTC	GGTCCCCATTTCCTTGGAACC
TCF15	TTCTTTGTGTAGGGACCGGGG	GGGCAGGCTGAATGGATCCT
GALNT3	CAGCAGAATTGAAGCCTGTCC	CTTCCCCACGTTCCTTTTCCT
GALNT5	GGGCGAGTCTTGGCATTTATC	CCCGAGTGTTGATCTCACTGAAT
GALNT7	GGTTCATCTTACGCAGTTTGCT	GGGCATGGGGTCATTGACA
GALNT10	TCAGCGCGTAGGAAATGGAG	GGAGAGAGCGATTCAAGGAGA
GCNT1	AACCCCTTAGTAAAGAAGAGGCG	AGCAGCCTGTCAAGCATTTCA
ST6GALNAC2	ACTTCCGTGGCCTGTTCAATC	GGCGATGACTTGGTGAGAGAG
RUNX2	TGGTTACTGTCATGGCGGGTA	TCTCAGATCGTTGAACCTTGCTA
COL1A1	GAGGGCCAAGACGAAGACATC	CAGATCACGTCATCGCACAAC
SOX9	AGCGAACGCACATCAAGAC	CTGTAGGCGATCTGTTGGGG
COL2A1	TGGACGATCAGGCGAAACC	GCTGCGGATGCTCTCAATCT
GAPDH	GGAGCGAGATCCCTCCAAAAT	GGCTGTTGTCATACTTCTCATGG
